# Wine, Beer, Alcohol and Polyphenols on Cardiovascular Disease and Cancer

**DOI:** 10.3390/nu4070759

**Published:** 2012-07-10

**Authors:** Sara Arranz, Gemma Chiva-Blanch, Palmira Valderas-Martínez, Alex Medina-Remón, Rosa M. Lamuela-Raventós, Ramón Estruch

**Affiliations:** 1 Department of Internal Medicine, Hospital Clínic, Institut d’Investigació Biomèdica August Pi i Sunyer, University of Barcelona, Barcelona, 08036, Spain; Email: sarranz@clinic.ub.es (S.A.); gchiva@clinic.ub.es (G.C.-B.); p.valderasm@ub.edu (P.V.-M.); 2 CIBER de Fisiopatologia de la Obesidad y la Nutrición, Instituto de Salud Carlos III, Cordoba, 14004, Spain; Email: amedina@ub.edu (A.M.-R.); lamuela@ub.edu (R.M.L.-R.); 3 Nutrition and Food Science Department, CeRTA, INSA Pharmacy School, University of Barcelona, Av. Joan XXIII s/n, Barcelona 08028, Spain

**Keywords:** wine, beer, alcohol, polyphenols, cardiovascular disease, cancer

## Abstract

Since ancient times, people have attributed a variety of health benefits to moderate consumption of fermented beverages such as wine and beer, often without any scientific basis. There is evidence that excessive or binge alcohol consumption is associated with increased morbidity and mortality, as well as with work related and traffic accidents. On the contrary, at the moment, several epidemiological studies have suggested that moderate consumption of alcohol reduces overall mortality, mainly from coronary diseases. However, there are discrepancies regarding the specific effects of different types of beverages (wine, beer and spirits) on the cardiovascular system and cancer, and also whether the possible protective effects of alcoholic beverages are due to their alcoholic content (ethanol) or to their non-alcoholic components (mainly polyphenols). Epidemiological and clinical studies have pointed out that regular and moderate wine consumption (one to two glasses a day) is associated with decreased incidence of cardiovascular disease (CVD), hypertension, diabetes, and certain types of cancer, including colon, basal cell, ovarian, and prostate carcinoma. Moderate beer consumption has also been associated with these effects, but to a lesser degree, probably because of beer’s lower phenolic content. These health benefits have mainly been attributed to an increase in antioxidant capacity, changes in lipid profiles, and the anti-inflammatory effects produced by these alcoholic beverages. This review summarizes the main protective effects on the cardiovascular system and cancer resulting from moderate wine and beer intake due mainly to their common components, alcohol and polyphenols.

## 1. Introduction

Since ancient times wine has been closely associated with diet, particularly in Mediterranean countries [1], and for many years, moderate and regular consumption of wine has been associated with health benefits, with no scientific basis. However, over the last two decades, several studies around the world have demonstrated that intake of alcoholic beverages produces positive effects on antioxidant capacity, lipid profile and the coagulation system [2], that may explain the reduction in the risk of cardiovascular disease (CVD) [3,4], overall mortality [5] and other diseases observed in moderate drinkers. By contrast, alcohol abuse or binge drinking has undoubtedly been related to a large number of medical, social and work related problems (negative effects), including the development of alcohol dependence syndrome, several chronic diseases (liver cirrhosis, cardiomyopathy, encephalopathies, polyneuropathy, dementia) and accidents which eventually lead to death [6,7,8].

Several cohort studies have pointed out that light-to-moderate alcohol consumers have an increased survival compared to abstainers [9]. Current evidence also suggests the protective effects of moderate drinking on cardiovascular events including coronary heart disease (CHD) [10], ischemic stroke [11], peripheral arteriopathy and congestive heart failure [12]. Positive effects have also been reported for moderate alcohol consumption on cellular aging damage, cognitive function and dementia. These effects have been observed in a variety of patients, including diabetics, hypertensive subjects and those with previous CHD.

Beneficial effects of moderate alcohol intake against atherosclerosis have been attributed to its antioxidant and anti-inflammatory effects, as well as to its actions on vascular function. In this framework, part of these effects may be attributed to polyphenols mainly contained in wine and beer, as these compounds exhibit antioxidant [13], anticarcenogenic [14], anti-inflammatory [15], hypotensive [16] or even anticoagulant properties [17].

Since the French paradox was described two decades ago [18], several studies have focused their attention on the components of red wine (mainly polyphenols and especially resveratrol) in order to explain the inverse association observed between moderate wine consumption and the incidence of CVD, as well as the different effects of the various types of alcoholic beverages (with or without polyphenols), thereby opening the debate of which type of alcoholic beverage is more cardioprotective than others.

Although the chemical constituents of grapes and wine vary, similar beneficial effects have been observed in different varieties of red wine, with white wine seeming to benefit the cardiovascular system to a lesser extent than red wine. The greater health benefits of red wine may be related to its higher polyphenolic content because of the distinctive production processes between red and white wine. 

The mechanisms responsible for the healthy effects of wine are extremely complex due to the many different pathways involved. Both alcohol and polyphenolic compounds have been extensively studied, despite the continued controversy as to which component is the most active [19]. The underlying mechanisms to explain these protective effects against CHD include an increase in high-density lipoprotein (HDL) cholesterol, a decrease in platelet aggregation, a reduction in the levels of fibrinogen and an increase in insulin sensitivity, which have been attributed to the ethanol content in wine. Other studies have provided evidence that wine exhibits beneficial properties which are independent of the presence of alcohol, and should be attributed to their polyphenolic content [20,21].

Similarly, the compounds found in beer have different biological activities demonstrated in enzymatic assays or cell cultures such as antioxidant [22], anticarcinogenic [23,24,25], anti-inflammatory [26], estrogenic [27] and even antiviral properties [28]. Different profiles of *in vitro* biological activity have been described for these compounds which, combined together, could have a synergistic effect. 

Therefore, the objective of this review is to summarize the main protective effects of moderate wine and beer consumption on CVD and cancer by way of their bioactive compounds. 

## 2. Polyphenolic Compounds in Wine and Beer

Red wine polyphenols are a complex mixture of flavonoids (such as anthocyanins and flavan-3-ols) and nonflavonoids (such as resveratrol, cinnamates and gallic acid). Flavan-3-ols are the most abundant, with polymeric procyanidins (condensed tannins) composing up to 50% of the total phenolic constituents [29]. These compounds act as potent antioxidants as they reduce low-density lipoprotein (LDL) cholesterol oxidation, modulate cell signaling pathways, and reduce platelet aggregation. Red wine contains more polyphenols than white wine (around 10-fold) because during the wine making process, red wine, unlike white wine, is macerated for weeks with the skin which is one of the parts of the grape with the highest concentrations of phenolic compounds [30]. The concentrations in red wine range from around 1.2 to 3.0 g/L (Table 1).

Both flavonoids and nonflavonoid phenolic compounds have been implicated in the protective effects of wine on the cardiovascular system. Nevertheless, the stilbene resveratrol has been one of the most extensively studied nonflavonoids as a critical constituent that contributes to the health benefits of red wine. In experimental studies, resveratrol exhibited both cardioprotective and chemopreventive effects, inhibiting LDL oxidation and platelet aggregation in animal studies. It also inhibits the growth of some tumor types and exhibits anti-inflammatory, antibacterial, antifungal, antiviral, neuroprotective, antiproliferative and anti-angiogenic activities [31,32]. However, the beneficial effects of moderate wine consumption may be attributed to the overall mix of all of its components and not to a specific action of one, such as resveratrol. Indeed, progress can be achieved in the field of the cardiovascular health effects of polyphenols when the one-dimensional antioxidant view of polyphenols is replaced by a view considering their multifaceted bioactivity, as polyphenols are versatile bioactives rather than mere antioxidants.

**Table 1:** Polyphenolic compounds in red wine.

Phenolic compounds	(mg/L) *		Phenolic compounds	(mg/L) *
**Anthocyanins**			Kaempferol	2.3
Cyanidin 3-*O*-(6′-acetyl-glucoside)	0.8		Kaempferol 3-*O*-glucoside	7.9
Cyanidin 3-*O*-glucoside	2.1		Myricetin	8.3
Delphinidin 3-*O*-(6′-acetyl-glucoside)	4.2		Quercetin	8.3
Delphinidin 3-*O*-(6′-*p*-coumaroyl-glucoside)	1.8		Quercetin 3-*O*-arabinoside	4.9
Delphinidin 3-*O*-glucoside	10.6		Quercetin 3-O-glucoside	11.4
Malvidin 3-*O*-(6′-acetyl-glucoside)	35.2		Quercetin 3-O-rhamnoside	11.5
Malvidin 3-*O*-(6′-caffeoyl-glucoside)	1.8		Quercetin 3-*O*-rutinoside	8.1
Malvidin 3-*O*-(6′-*p*-coumaroyl-glucoside)	19.5		**Hydroxybenzoic acids**	
Malvidin 3-O-glucoside	99.7		2,3-Dihydroxybenzoic acid	0.8
Peonidin 3-O-(6″-acetyl-glucoside)	4.7		2-Hydroxybenzoic acid	0.4
Peonidin 3-*O*-(6′-*p*-coumaroyl-glucoside)	5.2		4-Hydroxybenzoic acid	5.5
Peonidin 3-*O*-glucoside	8.2		Gallic acid	35.9
Petunidin 3-*O*-(6′-acetyl-glucoside)	5.7		Gallic acid ethyl ester	15.3
Petunidin 3-*O*-(6′-*p*-coumaroyl-glucoside)	3.9		Gentisic acid	4.6
Petunidin 3-*O*-glucoside	14.0		Protocatechuic acid	1.7
Pigment A	0.7		Syringic acid	2.7
Pinotin A	2.2		Vanillic acid	3.2
Vitisin A	3.1		**Hydroxycinnamic acids**	
**Dihydroflavonols**			2,5-di-*S*-Glutathionyl caftaric acid	28.6
Dihydromyricetin 3-*O*-rhamnoside	44.7		Caffeic acid	18.8
Dihydroquercetin 3-*O*-rhamnoside	9.7		Caffeoyl tartaric acid	33.5
**Flavanols**			Ferulic acid	0.8
(+)-Catechin	68.1		*o*-Coumaric acid	0.3
(+)-Gallocatechin	0.8		*p*-Coumaric acid	5.5
(−)-Epicatechin	37.8		*p*-Coumaroyl tartaric acid	11.8
(−)-Epicatechin 3-*O*-gallate	7.7		Sinapic acid	0.7
(−)-Epigallocatechin	0.6		**Hydroxyphenylacetic acids**	
Procyanidin dimer B1	41.4		4-Hydroxyphenylacetic acid	1.6
Procyanidin dimer B2	49.7		**Stilbenes**	
Procyanidin dimer B3	94.7		*d*-Viniferin	6.4
Procyanidin dimer B4	72.9		e-Viniferin	1.5
Procyanidin dimer B7	2.7		Pallidol	2.0
Procyanidin trimer C1	25.6		Piceatannol	5.8
Procyanidin trimer T2	67.1		Piceatannol 3-*O*-glucoside	9.5
Prodelphinidin dimer B3	1.1		Resveratrol	2.7
**Flavanones**			Resveratrol 3-*O*-glucoside	6.2
Hesperetin	0.5		**Hydroxybenzaldehydes**	
Naringenin	0.5		Protocatechuic aldehyde	0.5
Naringin	7.5		Syringaldehyde	6.6
**Flavonols**			**Tyrosols**	
Isorhamnetin	3.3		Hydroxytyrosol	5.3
Isorhamnetin 3-*O*-glucoside	2.6		Tyrosol	31.2

* Mean value of bibliographic data from Phenol-Explorer Database, Version 1.5.7 (INRA in collaboration with the Wishart Research Group) [33].

**Table 2:** Polyphenolic compounds in beer.

Phenolic compounds	(mg/L) *		Phenolic compounds	(mg/L) *
**Simple Phenols**			**Flavanones**	
Vinil-4-fenol	≤0.15		Isoxanthohumol	0.04–3.44
Vinil-4-guayacol	≤0.55		8-Prenilnaringenin	0.001–0.24
Etil-4-fenol	≤0.01		6-Prenilnaringenin	0.001–0.56
Isoeugenol	≤0.04		6-Geranilnaringenin	≤0.074
Tyrosol	≤40		Taxifolin	≤1.0
Propil-4-siringol	≤0.2		**Flavanols**	
2,3-Dihydroxy-guaiacyl propan-1-one	≤0.034		(+)-Catechin	≤5.4
**Phenolic acids**			(−)-Epicatechin	≤3.3
4-Hydroxyfenilacétic	≤0.65		Catechin gallate	5–20
Homovanillic	0.05		Epicatechin gallate	5–20
**Alquilphenols**			Procyanidin B3	≤3.1
3-Metilcatecol	≤0.03		Prodelphynidina B3	≤3.3
4-Etilcatecol	≤0.01		Prodelphynidina B9	≤3.9
4-Metilcatecol	≤0.02		Procyanidin C2	0.3
Vinil-4-fenol	≤0.15		**Flavonols**	
**Benzoic acid derivatives**			Kanpherol	16.4
3,5-Dihydroxybenzoic	0.3		Kanpherol-3-rhamnoside	≤1.0
2,6-Dihydroxybenzoic	0.9		Quercetin	≤10
2-Hydroxybenzoic	≤2.0		3,7-Dimetilquercetin	0.003
3-Hydroxybenzoic	≤0.3		Miricetin	0.007
4-Hydroxybenzoic	≤9.6		Quercetin 3-*O*-Arabinoside	0.006
Protocatecuic	≤0.3		Quercetina 3-*O*-Rutinoside	0.90
Vanillic	≤3.6		Quercitrin	≤2.3
Gallic	≤0.2		Isoquercitrin	≤1.0
Siríngico	≤0.5		Rutin	≤1.8
*o*-Vanillin	≤1.6		**Isoflavones**	
Siringic aldehyde	≤0.7		Daidzein	≤0.005
**Cinnamic acids**			Genistein	≤0.01
*p*-coumaric	≤1.2		Formononetin	≤0.004
*m*-Coumaric	≤0.2		Biochanin A	≤0.015
*o*-Coumaric	≤1.5		**Flavones**	
5-Caffeoilquinic	≤0.8		Apigenin	0.042
Caffeic	≤0.3		**α-acids (humulones)**	1.7
Ferulic	≤6.5		***Iso*-α-acids (*iso*-humulones)**	0.6–100
Sinapic	≤0.7		**Other polyphenols **	
**Chalcones**			Catechol	0.1
Xanthohumol	0.002–1.2		Pirogalol	0.3

* Mean value of bibliographic data published by Estruch *et al*. 2011 [34].

Beer is one of the most consumed alcoholic beverages around the world, being rich in nutrients such as carbohydrates, amino acids, minerals, vitamins and other compounds such as polyphenols. Hop (*Humulus lupulus* L.) is one of the raw materials of beer which serves as an important source of phenolic compounds. Dried hop cones contain about 14.4% of polyphenols, mainly phenolic acids, prenylated chalcones, flavonoids, catechins and pro-antocianidins [35]. Around 30% of polyphenols from beer comes from hops and 70%–80% originates from malt [36]. Moreover, hops provide a resin containing monoacyl phloroglucinols which become bitter acids during the development process of beer, such as α-acids (humulones) and iso-α acids. Table 2 details the bioactive compounds found in beer, mainly polyphenols. The structural classes of polyphenols in beer include simple phenols, benzoic acid derivatives and cinnamic acid, coumarins, catechins, di- and tri-oligomeric proanthocyanidins, prenylated chalcones and α- and iso-α acids derived from hops. 

Different profiles of *in vitro* biological activities have been described for these compounds which, in combination, exert a synergistic effect. However, to extrapolate these results and evaluate the *in vivo* physiological effects of beer consumption it is necessary to study their bioavailability in the body. The compounds found in beer have different biological activities demonstrated *in vitro* as antioxidant, anticarcinogenic, anti-inflammatory, estrogenic and antiviral. However, further studies in humans are needed to determine whether the plasma concentrations of these compounds, derived from moderate consumption of beer, have the same bioactivity observed *in vitro*.

## 3. Effects of Alcohol and Polyphenols on the Cardiovascular System

Although several epidemiological studies have shown a protective effect of moderate alcohol consumption on the incidence of cardiovascular events, the exact mechanisms involved are still not well known. Up to now, lower risk of myocardial infarction in both sexes has been related to the effects of moderate alcohol consumption on lipoproteins (HDL), coagulation (fibrinogen) and sensitivity to insulin (haemoglobin A1c) [11]. The modulation of circulating cholesterol is the best established protective factor of alcohol intake [37]. Except in people with liver impairment, alcohol ingestion raises serum HDL-cholesterol levels by incompletely understood pathways [38]. Inverse relations of HDL-cholesterol and CHD risk operate substantially via removal of lipid deposits in large blood vessels. HDL also binds with cholesterol in the tissues and may aid in preventing LDL cholesterol oxidation. The net effect is a reduction in the plaque building in walls of large blood vessels, such as coronary arteries. One study suggested that the major HDL subfractions, HDL_2_ and HDL_3_, were involved. While HDL_3_ may be strongly related to alcohol intake, both HDL fractions are related to lower CHD risk [39]. Nevertheless, a recent meta-analysis has questioned this hypothesis suggesting that HDL is not one of the most important intermediate variables in the possible causal pathway between moderate alcohol intake and CHD [40].

Since CHD is also considered a disease related to oxidative stress, several studies have analyzed the effects of different alcoholic beverages on oxidant status. In a cross-sectional study, alcohol consumption showed a direct relationship with the plasma concentration of *in vivo* oxidized low density lipoproteins (LDL) [41], while another study observed that the levels of the DNA oxidation marker 8-oxo-deoxyguanosine decreased with the amount of alcohol consumed [42]. The final effect probably depends on the total amount of alcohol consumed. Thus, in interventional studies a change in daily beer alcohol consumption from moderate-heavy to light intake lowered plasma F2-isoprostanes in healthy non-smoking men [43]. Ethanol metabolism can produce free radicals and reduce the levels of glutathione, the major cellular protection against oxidative stress. However, in addition to alcohol, wine and beer contain polyphenols which could confer beneficial health properties compared to other classes of alcoholic beverages [44,45]. In a recent study comparing the antioxidant effects of a polyphenol-rich alcoholic beverage (red wine) with those of a polyphenol-free alcoholic beverage (gin), it was observed that compared to gin, red wine reduced plasma superoxide dismutase activity and malondialdehyde levels, as well as increased lag phase time of LDL oxidation analysis, suggesting that red wine has greater antioxidant effects, probably due to its high polyphenolic content [46]. 

Triglycerides may play an independent role in the risk of CHD. A subset of heavy drinkers has a substantial increase in triglyceride levels, but this is infrequently seen in light to moderate drinkers [47]. Alcohol (ethanol) inhibits several promoters of clotting, including platelet stickiness and fibrinogen levels [38]. In addition, moderate alcohol consumption also affects the fibrinolytic system. In fact, it increases plasminogen activator inhibitor activity and reduces plasminogen activator activity in the postprandial period (five hours after eating), a fact that may explain the reduction in the early morning cardiovascular events observed in moderate drinkers who consume alcohol with dinner [48]. 

A recent meta-analysis of prospective observational studies has observed that moderate alcohol consumption lowers the risk of type 2 diabetes [49]. In addition, randomized clinical trials have also demonstrated that moderate alcohol intake has beneficial effects on insulin concentrations and insulin sensitivity in non-diabetic patients [50], suggesting that moderate alcohol consumption decreases the risk of CVD and type 2 diabetes by improving insulin sensitivity. On the other hand, the results of some experimental studies have suggested that moderate consumption of wine could also protect endothelial cells from injury produced by hyperhomocysteinemia [51], although further studies are needed on this issue.

The previously mentioned effects of ethanol, mainly those on lipoproteins and clotting factors seem to account for 50% of the beneficial effect of alcohol consumption in the prevention of atherosclerosis. However, to explain the totality of the antiatherosclerotic effects of alcohol it is necessary to resort to additional mechanisms.

Other studies have shown that alcohol and individual polyphenols modulate EC fibrinolytic protein (t-PA, u-PA, PAI-1, u-PAR and Annexin-II) expression at the cellular, molecular and gene levels to sustain increased fibrinolytic activity [52].

In summary, the mechanisms by which alcoholic beverages exert their actions involve lipid regulation and systemic anti-inflammatory effects. The alcohol component (ethanol) increases HDL-cholesterol levels, inhibits platelet aggregation, and reduces systemic inflammation. However, red wine provides additional benefits to those of other alcoholic beverages probably due to its higher polyphenolic content, by decreasing blood pressure, inhibiting the oxidation of LDL particles and other favorable effects on the cellular redox state, improving endothelial function, inhibiting platelet aggregation, reducing inflammation and cell adhesion and activating proteins that prevent cell death. These effects are weaker in the case of white wine or beer probably due to the lower concentration of polyphenols compared to red wine.

## 4. The Effects of Wine and Beer on the Cardiovascular System

### 4.1. Epidemiological Studies

In previous studies evaluating whether different alcoholic beverages protect against CVD, a J-shaped relationship was found for increasing wine consumption and vascular risk [53]. A recent meta-analysis including a parallel and separate evaluation of wine and beer consumption indicated a similar protective effect for beer and wine against cardiovascular risk [54]. On the contrary, no statistically significant association with vascular events was apparent for the intake of spirits, the type of alcoholic drink with the highest alcohol concentration and the lowest polyphenolic concentration, suggesting that the polyphenolic constituents found in wine or beer could be (mainly) responsible for the beneficial effect of alcoholic beverages on vascular events [55,56,57,58,59,60].

A great number of epidemiologic studies have evaluated the effects of the three main types of alcoholic drinks (wine, beer and distilled drinks) on the cardiovascular system. Some studies such as the Copenhagen City Heart Study [53] or that carried out in the east of France [61] found a high significant relationship between low or moderate wine consumption and a lower mortality by CVD, whereas in many other great epidemiologic studies, especially those based on the registries of the Nurse’s Health Study [62] or the Health Professionals Follow-up Study [11], no differences were found between the protective effects of moderate consumption of the different types of drink consumed. In the meta-analysis by Di Castelnuovo *et al*., 2010 [59], the authors analyzed the effects of the consumption of wine and beer on cardiovascular risk separately, concluding that the relative risk of associated vascular disease with wine consumption was of 0.68 (confidence interval 95% 0.59 to 0.77) compared with non-drinkers.

However, in other prospective studies, it has been observed that the moderate consumption of alcoholic drinks with a high alcoholic grade (liquors and distillates) also has a cardioprotector effect [63]. This fact explains that part of the beneficial effects of alcoholic beverages is largely due to ethanol, and not to the other specific components of each type of drink. Thus, the question as to whether the beneficial effects of alcoholic beverages depend on the alcoholic or non-alcoholic components of these beverages remains open and may not be answered on the basis of the results of epidemiological studies alone (see later). 

Finally, other confounding factors should be taken into account in the study of the relationship between alcohol consumption and health. As the incidence of coronary disease is low in men and women under 40 and 50 years of age, respectively, a recent study concluded that young adults who consume alcohol moderately also presented a significant reduction of the risk of coronary disease compared with abstainers, although the protective effects are lower than those observed in middle-aged adults or older adults [64].

Other studies have pointed out that the lower mortality and the reduced risk of ischemic cardiopathy observed in moderate drinkers could be due to other factors such as the maintenance of an uncommon lifestyle (Mediterranean diet) or due to genetic factors that also play an important role in the protection of moderate alcohol consumption against CVD [2]. These epidemiological studies have controlled both the classical vascular risk factors and other confounding factors, such as diet, exercise and certain demographic and psychosocial characteristics, leaving little doubt as to the validity of results from an epidemiological point of view. 

However, to obtain the best scientific evidence, the conclusions obtained from the results of meta-analyses or systematic reviews should only be based on randomized clinical trials. It would, therefore, be necessary to analyze a number of randomised studies with long-term interventions in which hard end-points (cardiovascular mortality, non-fatal acute myocardial infarction and non-fatal stroke) have been evaluated. In the absence of these studies, the findings of epidemiological studies cannot be considered definitive, especially after striking differences have been observed between the results of epidemiological and intervention studies, assessing, for example, the effectiveness of antioxidant vitamins on ischemic CHD [65].

For more than one decade it has been known that atherosclerosis is not only due to simple lipid accumulation in the arterial wall of certain zones of the vascular system, but rather, this is accompanied by a chronic inflammatory reaction of low intensity that contributes to the formation of atheroma plaques [66]. The biochemical and cellular mechanisms that lead to the beginning and progression of atherosclerosis have been widely studied [67]. This complex process involves the participation of very diverse cells (endothelial cells, smooth muscular cells, monocytes, lymphocytes and platelets), adhesion molecules (selectins, integrins and those pertaining to the super family of immunoglobulins), cytokines (interleukin (IL)-6, monocyte chemotactic peptide-1) and enzymes (metalloproteases). The first stage of atherosclerosis consists of the adhesion of monocytes and lymphocytes to the endothelium facilitated by adhesion molecules. Later, these cells migrate to the sub-endothelial space where they accumulate lipids and produce cytokines, growth factors and hydrolytic enzymes, which also induce a migration and proliferation of smooth muscle cells in this sub-endothelial space. The perpetuation of this process gives rise to the formation of atheroma plaque that becomes symptomatic when fissure (unstable plaque) is produced and induces the generation of thrombus on ulcerated plaque which, if not quickly degraded (fibrinolysis), may significantly occlude the vascular lumen and give rise to clinical events such as acute myocardial infarction or ischemic stroke [68]. Therefore, in order to know the role of alcohol consumption on the initiation and progression of atherosclerosis, the effect of alcohol on all the factors participating in the different stages of this process should be analyzed. 

### 4.2. Clinical Trials

As stated before, although there is general consensus concerning the lower risk for ischemic disease in moderate drinkers, there are discrepancies as to whether this cardioprotective effect is due to the ethanol in alcoholic beverages or to their non-alcoholic content, mainly polyphenolic compounds contained in some alcoholic beverages, especially wine or beer. Part of these issues may be solved only by the performance of well-designed randomized clinical trials analyzing the *in vivo* effects of wine and/or beer (in comparison with other alcoholic beverages). Randomized cross-over clinical trials will allow monitoring the diet of the subjects included and will eliminate the effects of antioxidants and other healthy compounds from the food, mainly fruits and vegetables. Up to now, the results of the different clinical trials have been contradictory. 

Thus, de Rijke *et al*. [69] examined the effect of the non-alcoholic components of red wine by reducing their alcohol content. The de-alcoholized red wine did not affect the susceptibility of LDL to copper-mediated oxidation. The results of this study did not show a beneficial effect of red wine consumption on LDL oxidation. By contrast, the study performed by Fuhrman, Lavy and Aviram [70] concluded that the consumption of red, but not white wine, by healthy volunteers reduced the propensity of LDL to undergo lipid peroxidation. However, some methodological problems related to the sample size, the sex of the subjects and dose or type of alcoholic beverage may be raised on analysis of these studies. In order to solve these issues, recent clinical trials have compared the effects of red wine, an alcoholic beverage with high polyphenol content, with gin, an alcoholic beverage without polyphenols. In this setting, Estruch *et al*. 2011 [46] showed that ethanol itself may exert significant beneficial effects on the cardiovascular system, mainly by increasing HDL-cholesterol and decreasing oxidation of LDL. However, red wine provided additional benefits due to its higher antioxidant effects, by decreasing serum malondialdehyde, blood superoxide dismutase activity and oxidized LDL plasma levels.

On the other hand, since atherosclerosis is considered an inflammatory disease, other studies have analyzed the effects of moderate consumption of wine and gin on inflammatory biomarkers [60,71]. Randomized clinical trials including wine and other alcoholic beverages without polyphenols showed anti-inflammatory effects by reducing plasma fibrinogen and IL-1α levels. Again, however, wine showed additional effects by decreasing highly sensitive C-reactive protein, as well as monocyte and endothelial adhesion molecules [60,72]. Related to the latter, new findings from a randomized, crossover trial developed with 67 high-risk male volunteers consuming red wine, the equivalent amount of de-alcoholized red wine, or gin, revealed that alcohol increased IL-10 and decreased macrophage-derived chemokine concentrations, whereas the phenolic compounds of red wine decreased serum concentrations of intercellular adhesion molecule-1, E-selectin, and IL-6 and inhibited the expression of lymphocyte function-associated antigen 1 in T-lymphocytes and macrophage-1 receptor, Sialyl-Lewis X, and C-C chemokine receptor type 2 expression in monocytes. Both ethanol and phenolic compounds of red wine downregulated serum concentrations of CD40 antigen, CD40 ligand, IL-16, monocyte chemotactic protein-1, and vascular cell adhesion molecule-1 [73]. Other studies performed in women observed that daily doses of 15**–**20 g of alcohol as red wine were sufficient to elicit anti-inflammatory effects similar to those observed in men who consumed higher doses of wine [74].

Many researchers have investigated the kinetics and extent of polyphenol absorption by measuring plasma concentrations and/or urinary excretion among adults after the ingestion of a single dose of polyphenol, provided as a pure compound or whole beverage. Subjects who showed higher absorption of polyphenols showed lower serum concentrations of inflammatory biomarkers than their counterparts [75]. Table 3 summarizes data from several relevant reviews about levels of polyphenolic metabolites in plasma and urine after wine or beer consumption. The results clearly show wide variability in the bioavailability of the different polyphenols. The polyphenols that are most well absorbed in humans are isoflavones and gallic acid, followed by catechins, flavanones and quercetin glucosides, with different kinetics. The least well-absorbed polyphenols are proanthocyanidins, the galloylated catechins and the anthocyanins. Data for other polyphenols are still too limited. Moreover, metabolism by the gut microbiota probably plays a major role in the biological activity of many polyphenols as in the case of prenylflavonoids of beer.

**Table 3:** Main polyphenolic metabolites described in literature from wine and beer.

Polyphenol group	Polyphenol metabolite	Source	Dose per day	No. subjects	Urine		Plasma		Ref.
					Urinary excretion	Tmax (h)	Mean Concentration	Tmax (h)	
Flavanols	Catechin	Red wine	35 mg (120 mL)	9		3.1	0.091 μmol/L	1.5	[76]
		Red wine	35 mg (120 mL)	9		3.2	0.077 μmol/L	1.44	[77]
		Red wine	35 mg (120 mL)	9	3%–10% of intake				[78]
	(+)-Catechin	Red wine	120 mL	9				1.6	[76]
	3′- *O*-Methylcatechin	Red wine	120 mL	9				1.2	[76]
Anthocyanins	Total anthocyanins	Red wine	218 mg (300 mL)	6	1.5%–5.1% of intake/12 h	6			[79]
	Malvidin 3-glc	Red wine	68 mg (500 mL)	6	0.016% of intake/6 h	<3	0.0014 μmol/L	0.83	[80]
Hydroxycinnamic acids	Caffeic acid	Red wine	0.9–1.8–2.7 mg (100, 200, 300 mL)	5			0.007–0.027 μmol/L	1	[81]
		Red wine	1.8 mg (200 mL)	10			0.037–0.060 μmol/L	0.5–1	[82]
		Red wine	55 μg/kg bw	12			0.084 μmol/L	2	[83]
		Red wine	5 mL/kg bw	12					[83]
		Beer	500 mL	10			0.05–0.07 μmol/L	1	[83]
	Ferulic acid	Beer	500 mL	10			0.11 μmol/L	1	[83]
	4-Hydroxyphenylacetic acid	Beer	500 mL	10		8	1.4–1.17 μmol/L	0.5–1	[84]
	Vanillic acid	Beer	500 mL	10			0.11 μmol/L	1	[84]
	*p*-Coumaric acid	Beer	500 mL	10			0.05–0.07 μmol/L	1	[84]
Hydroxybenzoic acids	Gallic acid	Red wine	4 mg (300 mL)	2			0.22 GA + 1.1 4-MeGA + 0.25 3-MeGA μmol/L		[85]
	Methylgallic acid	Red wine	47 μg/kg bw	12			0.18 4-MeGA μmol/L	2	[83]
	4- *O*-Methylgallic acid	Red wine	5 mL/kg bw	12					[83]
Stilbenes	*Cis*-resveratrol 3-sulfate	Red wine	250 mL	5	221.2 nmol/g creatinine	4			[86]
	Trans-resveratrol 3- *O*-glucuronide	Red wine	250 mL	5	179.2 nmol/g creatinine	4			[86]
	Trans-resveratrol 4′- *O*-glucuronide	Red wine	250 mL	5	59.6 nmol/g creatinine	4			[86]
	*Cis*-resveratrol 4′ sulfate	Red wine	250 mL	5	9294.2 nmol/g creatinine	4			[86]
	*Trans*-Resveratrol 3-sulfate	Red wine	250 mL	5	74.7 nmol/g creatinine	4			[86]
	*Trans*-Resveratrol 4′-sulfate	Red wine	250 mL	5	2.4nmol/g creatinine	4			[86]
	*Cis*-resveratrol 3-*O*-glucuronide	Red wine	250 mL	5	893.5 nmol/g creatinine	4			[86]
	*Cis*-resveratrol 4′-*O*-glucuronide	Red wine	250 mL	5	355.8 nmol/g creatinine	4			[86]

Tmax, time when Cmax is achieved; Cmax: maximal concentration; GA, gallic acid; MeGA, methylgallic acid; bw, body weight.

All these data provide strong biological plausibility to the epidemiological observations that moderate wine drinking reduces cardiovascular events. However, it should be taken into account that, although moderate wine intake reduces the risk of CHD, even low alcohol consumption in women may increase the risk of certain cancers, especially breast cancer [87].

In conclusion, moderate consumption of wine exerts a protective effect on biomarkers related to the progression and development of atherosclerosis due to its alcoholic (ethanol) and non-alcoholic (polyphenols) content. Women and those subjects with high polyphenol absorption are more sensitive to the healthy effects of wine.

Beer, despite its lower antioxidant and anti-inflammatory activity, also exerts a protective role against CVD. However, most studies addressing this issue are based on *in vitro* assays and animal studies, which have shown that the compounds derived from benzoic and cinnamic acids, catechins, procyanidins, humulones [23] and prenil-chalcones [88] are the major contributors to the antioxidant capacity of beer. It has also been observed that xanthohumol inhibited the oxidation of LDL *in vitro* induced by Cu^2+^ [89], as well as lipid peroxidation of liver microsomes in rats [90].

The anti-inflammatory mechanisms of bioactive compounds of beer are mainly due to the inhibition of inducible nitric oxide synthase (iNOS) and inhibition of the activity of cyclooxygenase 1 (COX-1) [23,26]. The main anti-inflammatory effect mediated by inhibition of iNOS induction seems to be due to xanthohumol. Moreover, xanthohumol and humulone produce an anti-inflammatory effect through inhibition of endogenous synthesis of prostaglandin E2 via COX-2 inducible by TNFα [23,26].

## 5. Role of Wine and Beer in Cancer Prevention

The consumption of alcohol has been identified as one of the top-10 risks contributing to the worldwide burden of disease. The International Agency for Research on Cancer (IARC) has classified ethanol as carcinogenic to humans [91]. Thus, the carcinogenic effects of alcoholic beverages are essentially due to their ethanol content and increase with the amount of alcohol drunk. Although the interpretation of data obtained in epidemiological studies is difficult due to confounding factors such as smoking, diet, hormone-replacement therapy and family history, the risks are essentially due to the ethanol content of alcoholic beverages. However, there is evidence that moderate wine consumption may decrease the risk of several cancers, including colon, basal cell carcinoma, ovarian and prostate cancer [92,93]. 

Consumption of approximately 1 glass of wine daily was associated with a decreased risk of developing Barrett’s esophagus, a precursor to esophageal adenocarcinoma, when compared to heavy drinkers or non-drinkers [94]. A meta-analysis found that modest wine consumption had an inverse association for developing lung cancer, for both average wine consumption of less than one drink per day (RR, 0.77; 95% CI, 0.59–1.00) and one drink or greater per day (RR, 0.78; 95% CI, 0.60–1.02) [95]. A study of female non-Hodgkin’s lymphoma patients found a significantly better five-year overall survival (75% *vs*. 69%) and five-year disease free survival (70% *vs*. 67%) in occasional wine drinkers *vs*. abstainers [96]. Compared to non-drinkers, women who drank wine for at least 25 years previously were 33% less likely to die and 26% less likely to experience a relapse or develop a secondary cancer over the five-year period following diagnosis. 

A large percentage of the literature on the cancer-preventing effects of wine are focused on one compound in particular: resveratrol. Many comprehensive reviews have been written on this subject [31,97,98]. Wine may inhibit carcinogenesis by acting as an antioxidant, anti-inflammatory agent, antimutagen, antimetastatic, anti-angiogenic, antidifferentiation, antiproliferative, and proapoptotic agent. It modulates signal transduction, immune response, transcription factors, growth factors, cytokines, caspases, interleukins, prostaglandin synthesis and cell cycle-regulating proteins.

However, although there are few doubts as to the beneficial effects of moderate drinking on the cardiovascular system, only a limited number of reports concerning alcohol and cancer have provided results in favor of a reduced risk of death by cancer. According to the data of the Million Women study [87] even light to moderate levels of alcohol consumption were predictive of an increased risk of several common cancers, mainly those of the breast. It has been suggested that alcohol intake increases circulating levels of estrogen and the production of reactive oxygen species during alcohol metabolism, inducing DNA damage that results in breast cancer [99,100]. A recent study has reported that alcohol intake, the genes involved in alcohol metabolism and their interaction, increase the risk of breast cancer in post-menopausal women [101]. In relation to the type of beverage (beer, wine or spirits), a prospective cohort study has reported that total alcohol intake of more than 27 drinks per week increases breast cancer risk in premenopausal women independently of the type of alcoholic beverage consumed [102]. However, a low to moderate alcohol intake was not associated with increased mortality after breast cancer in a cohort of middle-aged women previously diagnosed with breast cancer [103]. Additionally, the drinking pattern seems important in terms of alcohol-breast cancer association as some authors have suggested that low and regular wine consumption does not increase breast cancer risk [104].

Several components of wine (mainly resveratrol) have shown anticarcinogenic properties in experimental studies [105]. Those have been supported by its ability to inhibit proliferation of a wide variety of human tumor cells *in vitro* as well as implanted tumors, usually in mice [97,106]. However, it should be emphasized that these results are measurements of resveratrol responses on human cancer cells in culture, or taken as conclusions from epidemiological studies, rather than the results of clinical trials with cancer patients.

In the case of beer, xanthohumol is the best studied anticarcinogenic. It acts by inhibiting the metabolic activation of procarcinogenics, detoxifying enzyme inducers of carcinogens [25] and inhibiting tumor growth in early stages through inhibition of angiogenesis and inflammatory signals. Other compounds in beer with anticarcinogenic capacity are 8-prenilnaringenin, isoxanthohumol (having 10–20-fold lower concentrations than the effective doses in humans in beer) and other prenilflavonoids, as well as the flavanones, humulones and proantocianidins [24]. Considering that the bioavailability of the phenolic compounds of beer is very low, their anticarcinogenic effects are somewhat controversial as many epidemiological studies have shown [107,108]. However, polyphenols from beer can reach low but effective concentrations in the colon, acting as local anti-carcinogenic agents. The iso-α-acids (humulones) represent one of the most abundant groups of polyphenols in beer and also possess antitumor activity. It has also been described that cohumola, *n*-humulone and adhumulona activate receptors of α peroxisome proliferation (PPAR α), having potential activity in preventing cancer [23]. 

Several epidemiological studies have investigated the potential role of beer as a cause of cancer due to the detection of volatile nitrosamines in beer, although amounts have been reported to be lower in more recent decades because of changes in the beer-making process [109,110,111]. Since most epidemiological studies refer to long-term alcohol consumption, these studies cover, in part, periods of time before changes were made in the malting process. On the other hand, beer consumption (10 g alcohol/day) was reported to significantly decrease the risk of prostate cancer in comparison with non-drinking of beer in a Canadian study including 1253 subjects [112,113].

Malt-derived beer components require further investigation. Studies with melanoidins, *i.e.*, polymeric and colored final products of the Maillard reaction which are formed non-enzymatically during the roasting of malt, indicate peroxyl radical scavenging potential [114,115]. Melanoidin fractions with a relatively high molecular weight (10→200 kDa) also weakly induced NADPHcytochrome C reductase and size-dependently modulated GST activities in the Caco-2 colon cancer cell line [116]. The information available illustrates that beer is an extremely complex mixture of bioactive substances. Therefore, a thorough exploration of the chemopreventive activities of isolated prominent beer components seems to make eminent sense. Nevertheless, future studies should also focus on defined combinations to explore whether the mixture can be more efficacious than the single components.

Thus, the issue of alcohol and cancer is wide open and new studies are needed. Since self-reported data on alcohol consumption in epidemiological studies may not be reliable, especially in women, clinical studies on the effects of alcohol on health should be based on specific and accurate biomarkers of alcohol or wine/beer consumption such as ethanol (ethylglucoronide or ethylene glycol) or polyphenolic metabolites, respectively, in urine.

## 6. Conclusions

Sufficient evidence supports a significant inverse association between regular and moderate wine consumption and vascular risk, particularly red wine, and a similar relationship is reported for beer consumption, while lower protection is described for the consumption of any spirituous beverage.

Clinical and epidemiological studies indicate that it is mainly red wine which may protect against CVD, atherosclerosis, hypertension, certain types of cancer, type 2 diabetes, neurological disorders and metabolic syndrome. 

There is evidence that certain polyphenols, such as resveratrol, anthocyanins, flavonols and catechins in wine provide an abundance of health benefits. Furthermore, rather than polyphenols themselves, their metabolites might be the real key players in cardiovascular and cancer protection. In beer, xanthohumol and its metabolites isoxanthohumol and phytoestrogen 8-prenylnaringenin also provide healthy properties such as anticarcinogenic, anti-invasive, anti-angiogenic, anti-inflammatory and antioxidant effects. The complexity increases when considering that each subject may metabolize the beverage differently, making it impossible to establish one specific constituent as being critical from a health standpoint. 

It must be emphasized that the benefits associated with red wine and beer are dependent upon regular and moderate consumption. Although general recommendations are one drink (150 mL of wine or 10 g of alcohol) daily for women and two drinks (300 mL of wine or 20 g of alcohol) daily for men, individual ideals may vary based on age, gender, genetics, body type and drug/supplement use. These different recommended daily doses of alcohol between genders are explained by the fact that women are more sensitive to the effects of alcohol on the body. In addition, any healthy effects of wine and beer are greater in combination with a healthy diet. The health benefits associated with the Mediterranean diet, which combines moderate wine and beer consumption with a diet rich in fruits, vegetables and whole grains, suggests that polyphenols have synergistic effects with compounds found in other groups of foods. 

Although alcohol consumption is a two-sided coin, moderate alcohol consumption especially of wine has demonstrated the provision of a protective role for the cardiovascular system and in some types of cancer. Most medical professionals as well as the American Heart Association agree that heavy drinkers or alcohol abstainers should not be encouraged to drink wine for health reasons. Wine consumption should not replace a healthy lifestyle. However, light-to-moderate wine drinkers, without medical complications, may be assured that their wine consumption is a healthy habit.

Nevertheless, more randomized clinical trials focused on elucidating the mechanisms of the action of alcohol and polyphenols are needed.
